# Atom Transfer Radical Polymerization of Pyrrole-Bearing Methacrylate for Production of Carbonyl Iron Particles with Conducting Shell for Enhanced Electromagnetic Shielding

**DOI:** 10.3390/ijms23158540

**Published:** 2022-08-01

**Authors:** Miroslav Mrlík, Jozef Kollár, Katarína Borská, Markéta Ilčíková, Danila Gorgol, Josef Osicka, Michal Sedlačík, Alena Ronzová, Peter Kasák, Jaroslav Mosnáček

**Affiliations:** 1 Centre of Polymer Systems, University Institute, Tomas Bata University in Zlin, Trida T. Bati 5678, 760 01 Zlin, Czech Republic; ilcikova@utb.cz (M.I.); d_gorgol@utb.cz (D.G.); osicka@utb.cz (J.O.); msedlacik@utb.cz (M.S.); a_ronzova@utb.cz (A.R.); 2 Polymer Institute, Slovak Academy of Sciences, Dubravska Cesta 9, 845 41 Bratislava, Slovakia; jozef.kollar@savba.sk (J.K.); katarina.borska@savba.sk (K.B.); 3 Department of Physics and Materials Engineering, Faculty of Technology, Tomas Bata University in Zlin, Vavřečkova 275, 760 01 Zlin, Czech Republic; 4 Department of Production Engineering, Faculty of Technology, Tomas Bata University in Zlin, Vavřečkova 275, 760 01 Zlin, Czech Republic; 5 Center for Advanced Materials, Qatar University, Doha P.O. Box 2713, Qatar; peter.kasak@qu.edu.qa; 6 Centre for Advanced Material Application, Slovak Academy of Sciences, Dubravska Cesta 9, 845 11 Bratislava, Slovakia

**Keywords:** smart elastomer, polymer brushes, atom transfer radical polymerization, magnetic particle, interference shielding

## Abstract

The conducting polymer poly(2-(1H-pyrrole-1-yl)ethyl methacrylate (PPEMA) was synthesized by conventional atom transfer radical polymerization for the first time from free as well as surface-bonded alkyl bromide initiator. When grafted from the surface of carbonyl iron (CI) a substantial conducting shell on the magnetic core was obtained. Synthesis of the monomer as well as its polymer was confirmed using proton spectrum nuclear magnetic resonance (^1^H NMR). Polymers with various molar masses and low dispersity showed the variability of this approach, providing a system with a tailorable structure and brush-like morphology. Successful grafting from the CI surface was elucidate by transmission electron microscopy and Fourier-transform infrared spectroscopy. Very importantly, thanks to the targeted nanometer-scale shell thickness of the PPEMA coating, the magnetization properties of the particles were negligibly affected, as confirmed using vibration sample magnetometry. Smart elastomers (SE) consisting of bare CI or CI grafted with PPEMA chains (CI-PPEMA) and silicone elastomer were prepared and dynamic mechanical properties as well as interference shielding ones were investigated. It was found that short polymer chains grafted to the CI particles exhibited the plasticizing effect, which might be interesting from the magnetorheological point of view, and more interestingly, in comparison to the neat CI-based sample, it provided enhanced electromagnetic shielding of nearly 30 dB in thickness of 500 μm. Thus, SE containing the newly synthesized CI-PPEMA hybrid particles also exhibited considerably enhanced damping factor and proper mechanical performance, which make the material highly promising from various practical application points of view.

## 1. Introduction

Magnetorheological elastomers (MREs) are a special type of materials whose toughness can be tuned by application of an external magnetic field. Such smart elastomers (SE) generally belong to the class of matter called smart materials [1,2,3]. These are usually composed of two phases, filler and matrix, where the filler is in the most cases carbonyl iron (CI) particles possessing excellent magnetic performance (app. 200 emu/g) and the elastomeric matrix is mainly based on various on chemically cross-linked poly(dimethyl siloxane) (PDMS) [4,5] or synthetic rubbers such as styrene-butadiene rubber (SBR), acrylonitrile-butadiene rubber (NBR), etc. [6,7].

Next to the already-mentioned controllable toughness, other properties of such materials can also be achieved, such as magnetostriction or electromagnetic interference (EMI) shielding [8,9]. In this case, the proper dispergation of the particles is necessary and the off-state stiffness of the matrix also plays a crucial role in case of magnetostriction [10] or proper dielectric and magnetic properties are necessary in the case of electromagnetic shielding [11].

The electromagnetic shielding was already developed for various systems mainly based on magnetic particles and thermoplastic polymers [12] namely using poly(amide) 6 (PA6) [13] or using poly(vinylidene fluoride) and carbonyl iron [14]. However, such materials are rigid and thus have limitations in the deformation in comparison to elastomeric systems.

Therefore, there are various approaches to achieving the significant softening of the elastomeric systems, namely in the case of PDMS matrix, and they are described below. In order to reach controllable off-state stiffness of the matrix, in case of PDMS, the lower cross-linking density can be achieved by establishing the ratio between the monomer and cross-linker [10] or the addition of the silicone oil to the PDMS systems [15]. In the case of electromagnetic shielding, the addition of various conducting fillers to the MREs [16,17,18], utilization of the hybrid fillers based on magnetic particles [19,20,21] or development of core-shell particles mainly based on non-covalent modification [22,23] may be used.

Polypyrrole (PPy) has been widely used in the case of EMI shielding, although in the majority of cases as non-covalently bonded or as a part of the compound used for this purpose [16,24,25,26]. The covalent bonding of PPy is not possible, due to the fact that PPy is fabricated by oxidative polymerization. There were several trials to polymerize some pyrrole derivates such as n-vinyl pyrrole through the radical mechanism; however, in order to provide the conducting form, additional oxidation had to be performed [27,28]. Controllable modification of the CNT particles with pyrrole-based methacrylate was performed in 2017 by Radtke et al. [29] using electrochemical ATRP. Nevertheless, the confirmation of the controllable polymerization as well as on the narrow polymer chains distribution was missing.

Therefore, the main aim of this study was to prepare the magnetic particles covalently modified with conducting polymer. According to the best of our knowledge, we showed for the first time that poly(2-(1H-pyrrole-1-yl)ethyl methacrylate) (PPEMA) with controlled molar mass and narrow dispersity can be prepared via conventional atom transfer radical polymerization (ATRP). Surface-initiated ATRP under optimized conditions also enabled us to prepare the core-shell magnetic particles with a covalently bonded conducting polymer layer with controlled shell thickness in a single-step reaction. CI-PPEMA particles were properly characterized by various spectroscopy techniques and from the magnetic capability and electrical conductivity point of view. Finally, the mechanical performance and magnetorheological capability as well as electromagnetic shielding have been investigated for the most promising system.

## 2. Results and Discussion

### 2.1. SI-ATRP Grafting from the CI Surface

**Scheme 1:**
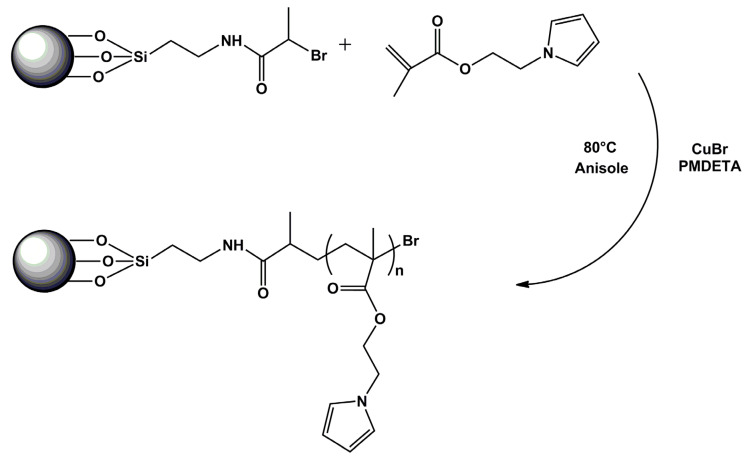
Schematic illustration of the SI-ATRP of the poly(1-H-pyrrole-1-yl)ethyl methacrylate (PPEMA) from the CI particles.

**Figure 1:**
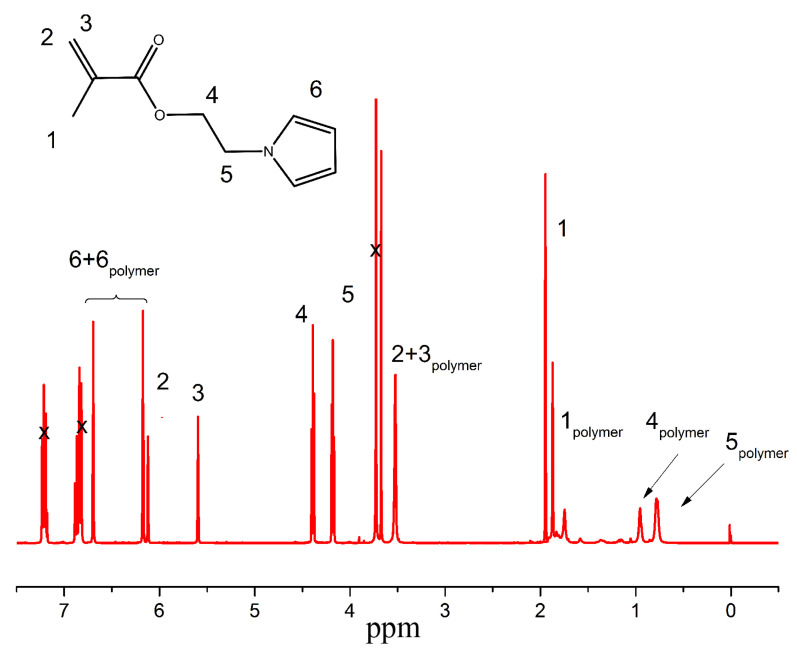
^1^HNMR spectra of the PPEMA polymer grafted from the surface of carbonyl iron. x representing the peaks of various solvents present in the reaction mixture.

**Figure 2:**
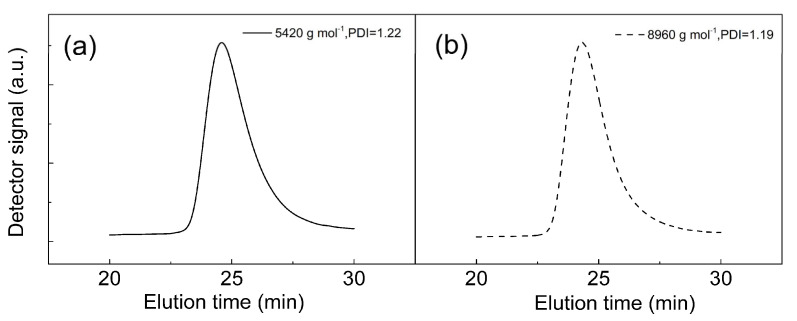
GPC analysis of the CI-PPEMA of various molecular weights (**a**) for 5420 g mol^−1^ and (**b**) for 8960 g mol^−1^.

The process of modification of the CI surface is schematically showed in Scheme 1. First, the CI surface was modified with an ATRP initiator by the reaction of the activated CI with silane coupling agent APTES and the subsequent amidation of amine groups with BiBB. Then, the SI-ATRP of PEMA from the CI-Br particles was performed. The polymer shell thickness onto the CI surface can be controlled by tailoring the molar mass of the polymer chains attached to the CI surface. The molar mass can be controlled by the monomer to initiator ratio and monomer conversion. In order to achieve good control of the molar mass, a sacrificial initiator, EBiB, was used in sufficient excess with respect to the expected amount of ATRP initiator covalently attached to the CI-Br surface. In addition, the use of the sacrificial initiator enabled the easy determination of molar mass by GPC and monomer conversion by NMR spectroscopy. The feed ratio of monomer to initiator was kept at 80:1 to tailor the shell of maximal theoretical thickness in a range of 5–20 nm. The polymerization was stopped after 6 h at 37% conversion (Figure 1). The molar mass of PPEMA was determined to be of 5420 g·mol^−1^ with a *Đ* of 1.22. In the case of reaction for higher molar mass, similar conditions were used; however, the monomer-to-initiator ratio was 160:1 and the polymerization was stopped after 10 h at 32% conversion. The molar mass was 8960 g·mol^−1^ with a *Đ* of 1.19 (Figure 2). The determination was based on the assumption of the similar growth of the polymer from the sacrificial and bond initiators [30]. A relatively low *Đ* implies a high polymerization control via ATRP, indicating good uniformity of the grafted polymeric layer. Due to the better conductivity of the iron particles achieved after controlled polymerization, the sample assigned as CI-PPEMA with a polymer chain length of 8960 g mol^−1^ was used for further physical investigations.

**Figure 3:**
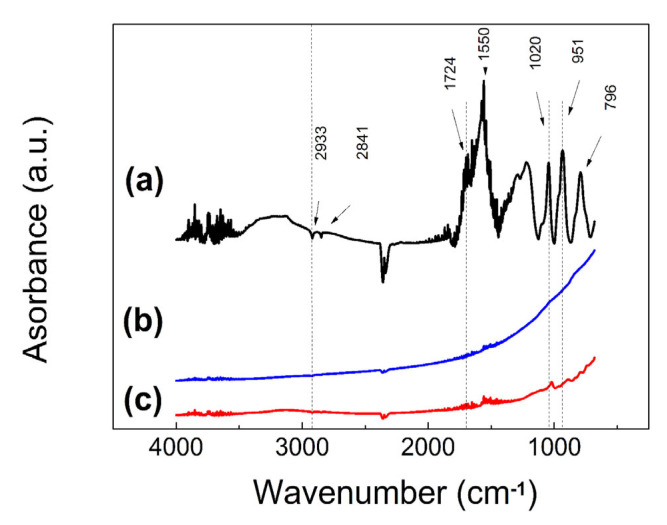
FTIR spectra of the PPEMA polymer (**a**), neat CI particles (**b**) and CI-PPEMA particles (**c**).

The presence of the grafted polymer on the surface of CI was confirmed by FTIR (Figure 3). As can be seen in Figure 3a, the typical C-H absorption bands are visible at 2993 cm^−1^ and 2841 cm^−1^. Furthermore, presence of carbonyl from methacrylate is visible for C=O stretching vibration at 1724 cm^−1^. Successful synthesis of PPEMA is also confirmed by peak at 1550 cm^−1^ as new absorption band of C-N. C-H vibrations are present at 1020 cm^−1^. The oxidized form of the PPy moiety is clearly seen at 951 cm^−1^, which is typical for certain doping state [31]. In case of CI-PPEMA composite particles, the most visible peaks corresponding to the carbonyl 1724 cm^−1^, to the C-H at 1050 cm^−1^ and finally to the oxidized form of PPy moiety present on the surface of the particles.

**Figure 4:**
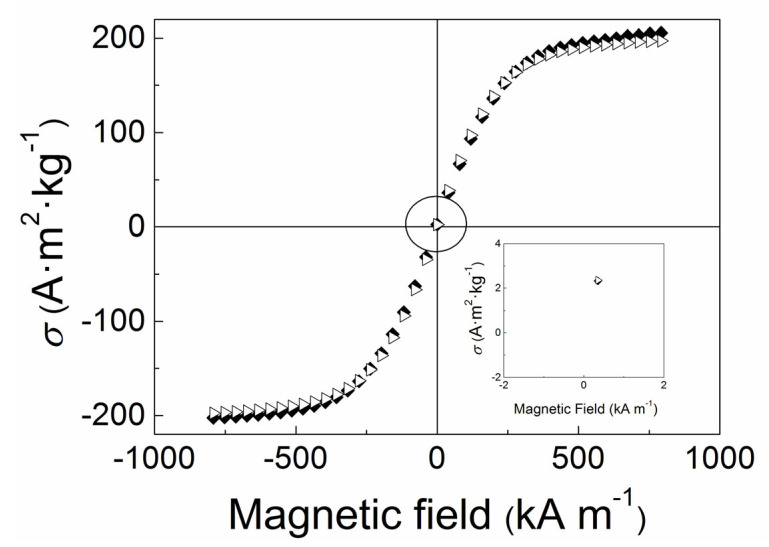
Magnetization curves of the bare CI (◆) and CI-PPEMA (▷) particles. The circle in the middle of the figure represents the figure inset position.

As can be seen in the Figure 4, the magnetization saturation of the bare CI particles is 198 emu/g and nearly negligible coercivity and magnetic remanence was measured. After covalent modification of the CI with PPEMA, the magnetization saturation just negligibly decreased to 192 emu/g probably due to the nano-size thickness of the PPEMA polymer layer, which was also seen in case of utilization of ATRP for modification of CI particles by different polymers in previous studies [10,32,33,34] or by modification using simple click chemistry [35,36]. Thus the magnetic properties are sustained on the same level, while the conductivity was considerably improved and was investigated further.

**Figure 5:**
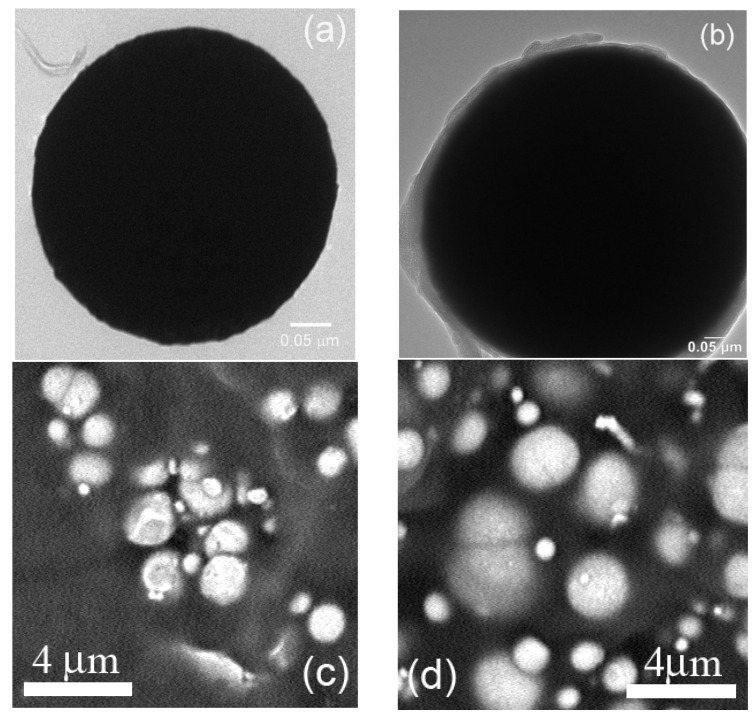

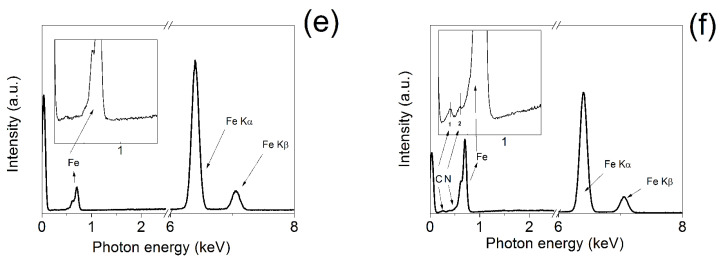
TEM images of the neat CI (**a**) and CI-PPEMA (**b**) particles. SEM images of the neat CI (**c**) and CI-PPEMA (**d**) particles in PDMS matrix and EDS spectra of the neat CI (**a**) and CI-PPEMA (**b**) particles. EDS of the neat CI (**e**) and CI-PPEMA (**f**). The numbers 1 and 2 in the figure (**f**) inset represents carbon and nitrogen elements, respectively.

As the pyrrole substituent on the controllably coated polymer shell was also partially oxidized during the ATRP polymerization, enhanced electrical conductivity has been obtained. The neat CI particles possessed 2 × 10^−3^ S cm^−1^, while the coated ones showed 6 × 10^0^ S cm^−1^. Generally, the poly(pyrrole) (PPy) powders shows in the doped state conductivities in the range from 0.1 to 10 S cm^−1^ [37]. Since the neat CI has three orders of magnitude lower conductivity, the major contribution to electric conductivity is therefore for the PPEMA shell. In order to confirm the successful grafting, TEM images (Figure 5) of both neat and modified CI particles were investigated. Neat CI has a typical spherical shape as was previously published [32] and CI-PPEMA particles have substantial coating around the whole magnetic particle. The diameter of the particles was not changed significantly from the average of 4 μm, since the layer seems to be no thicker than 50 nm. Moreover, the modification of the particles with PPEMA layer significantly improves the particle dispersion in the PDMS matrix. In the Figure 5c, the neat particles form the agglomerates in majority cases, while the CI-PPEMA particles are rather randomly and solely distributed within the matrix. The EDS investigation of the particles was also performed and is presented in the Figure 4e,f. It was confirmed that, in the case of neat CI particles, the spectra solely consist of Fe atoms signals, while in the case of CI-PPEMA, the particles contain also C and N elements, confirming the presence of the polymer layer grafted from the surface.

### 2.2. Dynamic Mechanical Analysis

The SE materials should have certain mechanical properties in order to be applicable in real life. Therefore, the dynamic mechanical analysis has been performed at a broad range of temperatures to see the changes after CI modification and investigate the impact of the grafting on the mechanical performance. 

**Figure 6:**
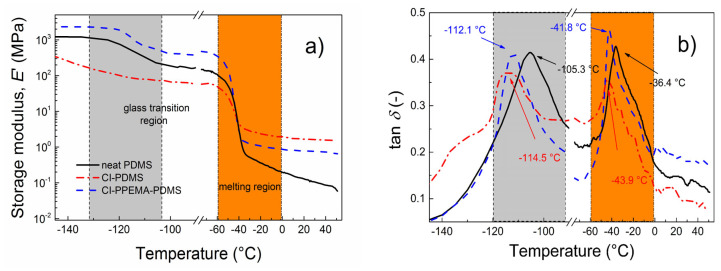
Temperature dependence of the tensile storage modulus, *E*′ (**a**) and damping factor, tan *δ* (**b**) for the neat PDMS matrix (*black solid lines*), the isotropic SE containing bare CI (*red dash/dot lines*), and CI-PPEMA (*blue dashed lines*) particles. The grey and orange region represent glass transition and melting, respectively.

As can be seen in Figure 6, the investigated samples exhibited temperature-dependent behavior typical for PDMS-based elastomers [38]. At low temperatures, below *T*_g_, the highest *E*′ was obtained for the SE containing CI-PPEMA particles (Figure 6a), which were well-dispersed in the PDMS matrix due to modification with a polymer shell using the ATRP approach, similarly to what was observed for other elastomeric systems with short polymer brushes [39,40]. On the other hand, the SE containing bare CI particles possessed the lowest *E*′, indicating the presence of agglomerates and thus the significant disintegration of this sample, which was pronounced at lower temperatures. Above *T*_g_, however, the situation was completely different. Around −40 °C, melting of the PDMS crystalline phase occurred (Figure 6), then at higher temperatures bare CI particles in the form of agglomerates possessed a strong reinforcing effect due to their possible covalent bonding with the PDMS matrix, which significantly enhanced the mechanical properties of the composite [41]. On the contrary, the SE containing CI-PPEMA particles exhibited, above the melting region, higher *E*′ values than the neat PDMS, but lower ones when compared to that of the SE containing bare CI. The results suggest that the mechanical properties of studied materials are strongly related to the crystalline phase of the PDMS matrix. Thus, the neat PDMS without any reinforcing agents (bare CI or CI-PPEMA) exhibited the lowest mechanical performance when the whole crystalline phase was melted. This is a crucial phenomenon, very frequently discussed for crystalline thermoplastic polymers such as poly(ethylene) [42], poly(propylene) [43] or poly(vinylidene fluoride) [44]; however, it is firstly addressed for PDMS elastomer in this article and taking into account that a potential applicability of such SE can be above the melting region.

The softening behavior was further clarified by the dependence of the tan *δ* on the temperature. As shown in Figure 6b, both SEs exhibited lower values of *T*_g_, namely –114.5 °C and –112.1 °C, for those containing bare CI and CI-PPEMA particles, respectively, when compared with the *T*_g_ value of the neat matrix (–105.3 °C) at a frequency of 1 Hz. Thus, the *E*′ of the SE containing CI-PPEMA was moderately lower and the tan *δ* was noticeably higher when compared to those of the SE containing bare CI. It was confirmed that the introduction of PPEMA grafts onto the CI particle surface moderately reduced *E*′, but largely enhanced the damping performance of the as-prepared, PDMS-based SEs; those can be used as a damping layers for various vibrations with controllable toughness.

### 2.3. Interference Shielding

**Figure 7:**
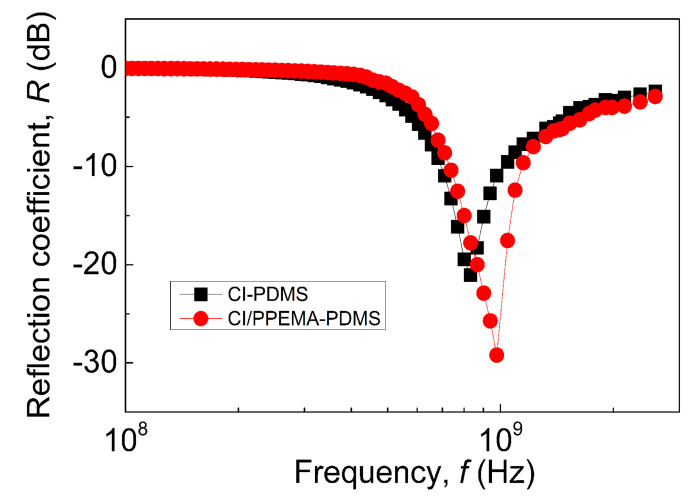
Electromagnetic shielding performance for neat CI based elastomers (■) and CI-PPEMA based elastomers (●).

As can be seen from Figure 7, SEs exhibit good absorbing ability at ultra-high frequencies with bandwidths ranging from 1.15 to 1.44 GHz and a maximum value of reflection coefficient of nearly 30 dB for CI-PPEMA-based PDMS composite with a sample thickness of 500 μm. In this case, we are assuming a single-layer metal-backed absorber model (Equation (1)), i.e., the reflection of the acting wave. Moreover, the electromagnetic shielding materials based on CI-PPEMA particles have different operating frequency ranges comparing to SE based on pristine CI particles. Thus, it is possible through the modification of the particle surface and thereby affecting the frequency characteristics of permeability and permittivity of MREs to tune the operating frequency range of SE, making possible their application in the frequency range from nearly 700 MHz to 1.6 GHz, in which many communication and information transmission systems usually operate [9]. The values presented in this study are very promising and still competitive to the state of the art as reported by Anju et al. combining magnetic nanoparticles, reduced graphene oxide in TPU matrix with shielding efficiency on transmission of 58.8 dB for a sample thickness 1 mm [45]. Utilization of the magnetite particles with reduced graphene oxide provides the reflection of 25 dB with a sample thickness of 1 mm [46]. Moreover, the application of the nanoscale iron/iron-carbide graphite particles in the PVDF matrix significantly improve the EMI shielding, reaching a reflection coefficient of 40.5 dB for a 4.3 mm-thick specimen. Another set of already published systems are summarized in Table 1. Our designed system has significant benefit in the core-shell particle system, when the core is magnetic and the shell is electrically conductive and covalently bonded to the core. Therefore, the processing of our hybrid particles is advantageous since the distribution of the magnetic and electrically conducting particles is homogenous. In the case of utilization of individual graphene oxide particles, they could even be reduced, as such homogenous distribution is always a problem and was not considered in the mentioned studies.

## 3. Methods and Materials

### 3.1. Materials

Carbonyl iron (CI) BASF, ES grade has min. 97% iron content. Silicone elastomer Sylgard 184 (Dow Corning, MI, USA) and silicone oil M100 (Kolín, Czech Republic) were used as received, along with 2-choloroethanol (EtOCl, 97%), pyrrole (Py, 98%) methacryloyl chloride (98%), Aminopropyl(triexthosy) silane, coupling agent (APTES, 97%) and Initiator α-bromoisobutyryl bromide (BiBB, 98%). Initiator bonding was performed in the presence of proton scavenger, triethylamine (TEA, ≥99%), ethyl α-bromoisobutyrate (EBiB, 98%), N,N,N′,N″,N″-pentamethyldiethylenetriamine (PMDETA, ≥99%), copper bromide (CuBr, ≥99%) and anisole (99%) were used as a monomer, initiator, ligand, catalyst and solvent, respectively. Diethyl ether (ACS reagent, anhydrous, ≥99%) was used as a drying agent. All chemicals were purchased from Sigma Aldrich (St. Louis, MO, USA) and were used without further purification. Tetrahydrofuran (THF, p.a.), acetone (p.a.), ethanol (absolute anhydrous, p.a.), toluene (p.a.), and potassium hydroxide (KOH, p.a.) were obtained from Penta Labs (Prague, Czech Republic). Deionized water (DW) was used during all experimental processes and washing routines.

### 3.2. Initiator Bonding

The CI particles has been covalently modified with APTES according to the procedure described elsewhere [10]. The NH_2_ functional groups present on the surface after APTES modification were used as active sites for attachment of BiBB molecules. In a simple procedure, the CI (5 g), dried THF (60 mL) and TEA (12 mL) were mixed under argon atmosphere at a temperature of ~5 °C ensured by an ice/water bath, while BiBB (10 mL) was added drop by drop. The product was washed with THF, acetone and water several times and then decanted using permanent magnet. The excess water from treated particles was removed by vacuum drying at 60 °C overnight.

### 3.3. Synthesis of 2-(1H-Pyrrole-1-yl)ethyl Methacrylate Monomer

The process is a two-step synthesis; firstly, freshly distilled pyrrole reacts with 2-chloroethanol. The synthesis was performed as follows: a magnetic stirrer and KOH were evacuated for 30 min in a three-neck flask and backfilled with argon. Dried DMSO (100 mL) was added under argon atmosphere. Freshly distilled pyrrole (4 mL) was bubbled with argon for 15 min and added to the mixture. Finally, 2-chloroethanol (1.6 g) bubbled for 30 min with argon was added dropwise at velocity of 4 mL/hour. The reaction was carried out for 12 h at ambient temperature. The mixture was extracted with dichloromethane and brine 3 times and dried over sodium sulfate. Then, the column extraction was performed with eluent isohexane:ethyl acetate in a ratio of 3:2, then again column extraction in isohexane:ethyl acetate in ratio 2:1 and pure product, 2-(1H-pyrrole-1-yl)ethanol (HEP) (yellowish liquid) after evaporation of the solvent excess using vacuum rotator. The yield of this reaction was calculated as 75%. The product was dissolved in CDCl_3_ and characterized using nuclear magnetic resonance (NMR) ^1^H NMR (CDCl_3_): *δ* 6.70 (dd, C_4_H_4_N), 6.18 (dd, C_4_H_4_N), 4.07 (t, NCH_2_CH_2_), 3.84 (t, HOCH_2_CH_2_), 1.75 (s, COH).

**Scheme 2:**
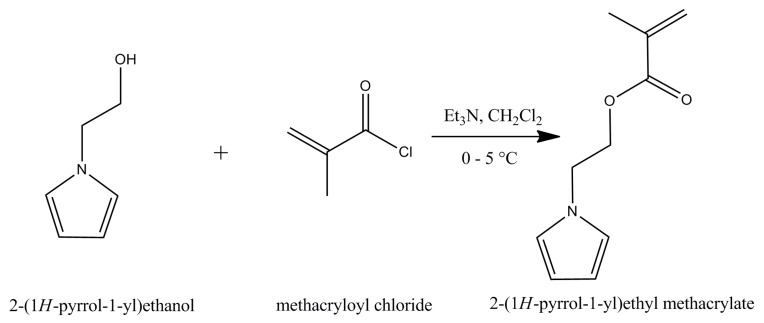
Schematic illustration of the monomer synthesis.

**Figure 8:**
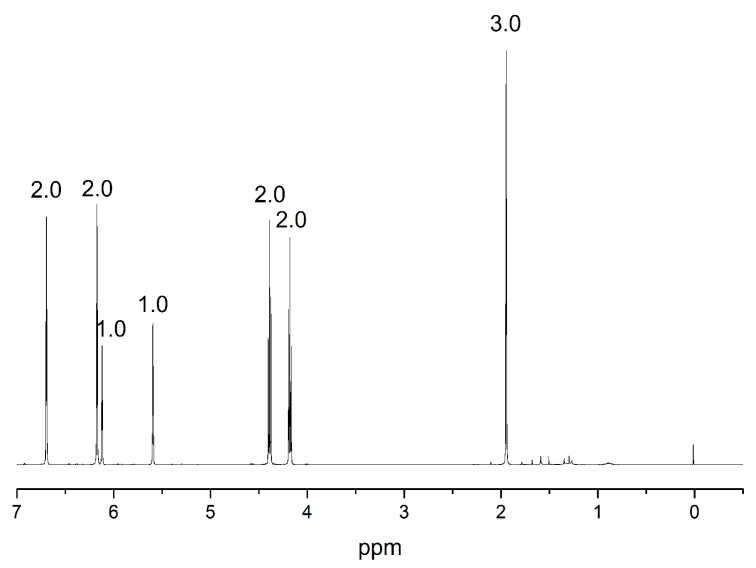
^1^HNMR spectrum of the 2-(1H-pyrrole-1-yl)ethyl methacrylate (PyEMA).

In the second step, the reaction was carried out in the 3-neck flask equipped with magnetic stirrer evacuated for 30 min and backfilled with argon. Dried dichloromethane (20 mL) was added to the mixture under argon atmosphere. HEP (1.5 g) with TEA (2.8 mL) were bubbled for 15 min with argon and added to the mixture and the temperature was maintained for 1 h between 0 and 5 °C. Freshly distilled methacryloyl chloride (1.6 mL) was added dropwise at a velocity of 5 mL/h. This is schematically shown in Scheme 2. Then mixture was further maintained for 2 h between 0–5 °C. The reaction was carried out for another 14 h at ambient temperature. The first extraction was performed with dichloromethane to the water 3 times, in order to dissolve created ammonium salt. The second extraction was performed with dichloromethane with the brine 3 times to purify the product and it was then dried with sodium sulfate. The product was obtained after evaporation of solvent excess with vacuum rotator. Finally, the product was cleaned through neutral alumina using dichloromethane (Figure 8). The yield of this reaction was calculated at 72%.

### 3.4. Surface Initiated ATRP Polymerization of 2-(1H-Pyrrole-1-yl)ethyl Methacrylate (PyEMA) from the CI Particles

In order to prove that prepared monomer PyEMA can be synthesized using the ATRP approach, the following general procedure was utilized. A Schlenk flask (SF) equipped with magnetic particles (1 g) was evacuated several times and then backfilled with argon. Then, monomer PyEMA (3 g 13 mmol), PMDETA (0.035 mL; 0.16 mmol), EBiB (0.025 mL; 0.16 mmol) and anisole (3 mL; 50 vol. %) were stepwise injected into the SF under argon atmosphere. Several freeze-pump-thaw cycles with liquid nitrogen were performed to eliminate residual oxygen from the polymerization mixture, and finally, the flask was filled with argon. The polymerization was initiated by addition of CuBr catalyst (0.023 g; 0.16 mmol) to the polymerization mixture and placing the reaction flask into the glove box with inert argon atmosphere and with the oil bath pre-heated to 80 °C. The reaction mixture was stirred at 250 rpm for 6 or 10 h and then the reaction was stopped by removing the flask from the glove box and aerating.

### 3.5. Fabrication of the Neat SE and SE Containing Neat and SI-ATRP Modified CI Particles

Silicone elastomer, silicone oil and cross-linking agent were mixed in a ratio of 7:3:1, respectively. The mixture was placed to the cylindrical form with diameter 25 mm and 500 μm thickness. Then, they were put into the oven preheated for 40 °C for 6 h to reach high conversion of cross-linking.

#### 3.5.1. Characterization

^1^H nuclear magnetic resonance (NMR) spectra were recorded at 25 °C using an instrument (400 MHz VNMRS Varian, Tokyo, Japan) with deuterated chloroform (CDCl_3_) as a solvent. The molar mass and dispersity (Đ) of PPEMA chains were investigated using gel permeation chromatography (GPC) on the GPC instrument (PL-GPC220, Agilent, Tokyo, Japan) equipped with GPC columns (Waters 515 pump, two PPS SDV 5 μm columns (diameter of 8 mm, length of 300 mm, 500 Å + 105 Å)) and a Waters 410 differential refractive index detector tempered to 30 °C. The samples for GPC analysis were prepared by their dilution with THF, followed by the purification process, in which they were passed through a neutral alumina column. The thickness of the grafted layer was observed using a transmission electron microscope (TEM, JEM-2100Plus, Jeol, MA, USA). The samples for TEM analysis were prepared by dispersing the particles in acetone and a dropping onto a copper grid. Fourier-transform infrared (FTIR) spectra (64 scans, resolution of 4 cm^−1^) were recorded on a Nicolet 6700 (Nicolet, Glendale, WI, USA) within a wavenumber range of 3600–600 cm^−1^, while the ATR technique with a Germanium crystal were employed. The spectra were recorded at room temperature and sample was in the form of the pellet. The powders were compressed to the form of pellets (diameter of 13 mm, thickness of 1 mm) on a laboratory hydraulic press (Trystom Olomouc, H-62, Olomouc, Czech Republic). The pellets were used for electrical conductivity measurements, which were performed by the two–point method at laboratory temperature with the help of an electrometer (Keithley 6517B, Cleveland, OH, USA). The magnetic properties of the neat CI particles as well as CI-PPEMA were studied using a vibrating-sample magnetometry (VSM) (Model 7404, Lake Shore, MA, USA) in the magnetic fields approaching to ±1000 kA/m at laboratory conditions. The samples in the form powders were accommodated into the VSM sample holder (730931 Kel-F, powder/bulk upper/bottom cup). 

Interference shielding was evaluated using complex magnetic permeability and dielectric permittivity of SE with randomly distributed particles (30 vol.%) of both types, coated and uncoated in silicone matrix, have been measured at room temperature in the frequency range of 1 × 10^7^–3 × 10^9^ Hz by the impedance method with an impedance/material analyzer Agilent E4991A (Agilent Technologies, Tokyo, Japan). The investigation of dielectric properties was carried out on circular samples with diameters of 15 mm, whereas the measurements of complex magnetic permeability were performed on toroidal samples with an outer diameter of 8 mm and an inner diameter of 3.1 mm. Specimens for both characterizations were cut out of previously prepared plates (thickness 500 μm) by a manual press. In order to estimate the absorbing properties of single-layer metal-backed ESMs based on investigated SE, the frequency dependence of the reflection coefficient, R, representing the absorbing ability of ESMs in decibels, has been calculated. When the level of R is equal to −10 dB and the absence of transmitted energy is presumed, then 90% of absorption of incident energy by ESM is obtained. Given that an electromagnetic wave is incident on the ESM surface along the normal, R from the surface of such a material can be calculated according to Equations (1)–(3) present in this paper, those are more precisely described in the paper Lopatin et al. [21,49]:

(1) $$R_{L}\left(\mathrm{dB}\right)=20\log\left|\frac{Z_{in}-1}{Z_{in}+1}\right|$$

where

(2) $$Z_{in}=\sqrt{\frac{\mu^{*}}{\varepsilon^{*}}\tanh\left(j\frac{\omega }{c}\sqrt{\mu^{*}\varepsilon^{*}d}\right)}$$

is the input impedance of the ESM, *c* is the velocity of light, *ω*(=2 pf) is the angular frequency, μ* = *μ*′ − *jμ*″ and ε* = *ε*′ − *jε*″ are the complex permeability and permittivity of the material, respectively, and *d* is the thickness of the sample. The reflection from ESM is absent in case *Z_in_* = 1. However, the absence of reflection from the ESM in real materials is reached only approximately, and the frequency, *f*_0_, and thickness, *d*_0_, for which the above condition is satisfied with the highest degree of accuracy, are called the matching frequency and matching thickness, respectively. In practical calculations, the minimum of *R* is obtained only for complex values of thickness:

(3) $$d=d^{\prime }+jd^{\prime \prime }=\frac{c}{2\pi f\sqrt{\mu^{*}\varepsilon^{*}}\arctan}\left(-j\sqrt{\frac{\varepsilon^{*}}{\mu^{*}}}\right)$$

Once the dependence of the complex parameter *d* (Equation (3)) on frequency is calculated, the minima satisfying the inequality (*d*″/*d*′) ≥ 0.01 are taken and the thickness *d*_0_ = *d*_0_′ is substituted into Equations (1) and (2) [21], yielding the frequency dependence of *R*. The minus sign for the reflection coefficient expressed in decibels is again given by Equation (1), where the value is logarithmic by a decimal logarithm less than 1. However, for reasons of simplicity and ease of comparison, the absolute value of the determined reflection coefficient can be taken.

#### 3.5.2. Dynamic Mechanical Analysis

A dynamic mechanical analysis (DMA) in tensile mode was performed on a DMA/SDTA816e (Mettler Toledo, Curych, Switzerland). The tested samples were in the form of strips with dimensions of 15 mm in length, 1.8 mm in width and 1.15 mm in thickness cut from the sample specified in part 2.5. All measurements were performed in the LVR determined from the strain dependence of tensile storage modulus, E′. Temperature sweeps were examined in a temperature range from −145 to 50 °C at a heating rate of 3 °C·min^−1^ at different frequencies under a nitrogen atmosphere. The conditions were chosen in order to properly investigate the *E*′ evolution and the glass transition temperature, *T*_g_, as a peak position of tan *δ*. The samples were measured twice, and average values were used for further evaluation.

## 4. Conclusions

In this study, a novel approach to synthesizing particles possessing both magnetic and electric activity was developed. The magnetic particles of CI were for the first time controllably coated with conducting pyrrole-based polymer PPEMA. First, the monomer PEMA was synthesized and characterized by ^1^HNMR. Then, the homopolymerization was optimized and final product was characterized using GPC and conductivity measurement. Successful grafting and influence of the CI particles on the polymerization were investigated and confirmed by FTIR, TEM and GPC, respectively. Negligibly affected magnetic properties, due to the very thin covalently coated polymer layer, were confirmed using VSM. Presented surface modification is beneficial in comparison to randomly coated systems due to the possibility of controllable targeting of the thin, electrically conductive shell. Final properties of CI-PPEMA, such as mechanical properties, were correlated with bare CI in order to see the possibility of application in real life and it was shown that damping was considerably enhanced. The interference shielding investigations proved the improved capability of the developed system in electromagnetic shielding at high frequencies and reached nearly 30 dB with a sample thickness only of 500 μm. The achieved high shielding at low thickness allows fewer requirements regarding the amount of material for such systems in comparison to the state of the art.

## Figures and Tables

**Table 1:** EMI shielding characteristics of polymer magnetic composites reported in some previous literatures.

Polymer Matrix	Filler	Thickness (mm)	Shielding (Reflection) (dB)	Frequency Band (GHz)	Ref. (DOI)
TPU	CuCoF1/rGO	1.0	19.8	8.2–12.4	[45]
PA	NiFe_2_O_4_/rGO	2.0	13.4	8.2–12.4	[47]
PS	rGO/ CoFe_2_O_4_	1.9	48.4	0.1–20	[48]
TPU	CI/TEOS	0.5	18.0	0.1–3	[9]
PDMS	CI	0.5	21.1	0.1–3	This work
PDMS	CI/PPEMA	0.5	29.9	0.1–3	This work

## Data Availability

Data used in this manuscript are available upon request of the corresponding authors.
